# Gauging megadiversity with optimized and standardized sampling protocols: A case for tropical forest spiders

**DOI:** 10.1002/ece3.2626

**Published:** 2016-12-20

**Authors:** Jagoba Malumbres‐Olarte, Nikolaj Scharff, Thomas Pape, Jonathan A. Coddington, Pedro Cardoso

**Affiliations:** ^1^ Center for Macroecology, Evolution and ClimateNatural History Museum of DenmarkUniversity of CopenhagenCopenhagenDenmark; ^2^ Natural History Museum of DenmarkUniversity of CopenhagenCopenhagenDenmark; ^3^ Smithsonian InstitutionNational Museum of Natural HistoryWashingtonDCUSA; ^4^ Finnish Museum of Natural HistoryUniversity of HelsinkiHelsinkiFinland

**Keywords:** Araneae, Arthropoda, optimization algorithm, rapid biodiversity assessment, sampling methodology, species richness, Udzungwa Mountains

## Abstract

Characterizing and monitoring biodiversity and assessing its drivers require accurate and comparable data on species assemblages, which, in turn, should rely on efficient and standardized field collection. Unfortunately, protocols that follow such criteria remain scarce and it is unclear whether they can be applied to megadiverse communities, whose study can be particularly challenging. Here, we develop and evaluate the first optimized and standardized sampling protocol for megadiverse communities, using tropical forest spiders as a model taxon. We designed the protocol COBRA‐TF (Conservation Oriented Biodiversity Rapid Assessment for Tropical Forests) using a large dataset of semiquantitative field data from different continents. This protocol combines samples of different collecting methods to obtain as many species as possible with minimum effort (optimized) and widest applicability and comparability (standardized). We ran sampling simulations to assess the efficiency of COBRA‐TF (optimized, non‐site‐specific) and its reliability for estimating taxonomic, phylogenetic, and functional diversity, and community structure by comparing it with (1) commonly used expert‐based ad hoc protocols (nonoptimized, site‐specific) and (2) optimal protocols (optimized, site‐specific). We then tested the performance and feasibility of COBRA‐TF in the field. COBRA‐TF yielded similar results as ad hoc protocols for species (observed and estimated) and family richness, phylogenetic and functional diversity, and species abundance distribution. Optimal protocols detected more species than COBRA‐TF. Data from the field test showed high sampling completeness and yielded low numbers of singletons and doubletons. Optimized and standardized protocols can be as effective in sampling and studying megadiverse communities as traditional sampling, while allowing data comparison. Although our target taxa are spiders, COBRA‐TF can be modified to apply to any highly diverse taxon and habitat as long as multiple collecting techniques exist and the unit effort per sample is comparable. Protocols such as COBRA‐TF facilitate studying megadiverse communities and therefore may become essential tools for monitoring community changes in space and time, assessing the effects of disturbances and selecting conservation areas.

## 1 Introduction

Inventorying and characterizing megadiverse communities is an overwhelming but necessary task (Basset et al., 2012; Blackmore, 1996; Coddington & Levi, 1991; Colwell & Coddington, 1994; Lawton et al., 1998; Magurran & Queiroz, 2010). Comprehensive descriptions of the structure and dynamics of these communities are critical to mitigate processes such as defaunation (Dirzo et al., 2014). Improving sampling protocols and analytical methods for biodiversity assessment and monitoring is one of the main priorities in arthropod research and conservation (Cardoso, Erwin, Borges, & New, 2011; Didham, Basset, & Leather, 2010), and standardized protocols are therefore an important tool (Cardoso, 2009; Cardoso, Crespo, Carvalho, Rufino, & Henriques, 2009; Cardoso, Erwin et al., 2011).

Standardized sampling provides species richness and relative abundance data that enables comparison of assemblages even with incomplete species lists, allowing for statistical inference in ecology, biogeography, and other fields (Duelli, 1997; Duelli, Obrist, & Schmatz, 1999; Jones & Eggleton, 2000; Stork, Samways, & Eeley, 1996). The paucity of standardized sampling protocols and the consequent lack of data are some of the reasons why arthropods are often neglected in conservation programs (Cardoso, Erwin et al., 2011; New, 1999; Stork et al., 1996). Standard protocols exist for large‐scale or even global inventories of ants (Agosti, Majer, Alonso, & Schultz, 2000) and butterflies (Pollard & Yates, 1993), for carabid beetles in environmental monitoring (Niemelä et al., 2000), and for stream macroinvertebrates (Hering, Moog, Sandin, & Verdonschot, 2004), among others.

We find it useful to view the problem of designing standardized sampling protocols as comprised of seven complementary criteria (Cardoso, 2009; Chao & Jost, 2012). The first is efficiency: Does the protocol have high return (of important data) on investment (time and resources required to acquire the data)? The second is suitability for a specific taxon or problem: Sampling in cloud forests with high epiphytic biomass may require a different protocol compared to lowland dry forest or rain forest—and closed canopy tropical forests may be different from, say, savannas or Mediterranean ecosystems. Suitable for one problem may not be suitable for another. The third is comparability: If the second criterion, suitability, is the only priority, one may end up with results that cannot be compared between sites, habitat types, or biomes, even if broadly similar. Comparability ideally means that one protocol design can be applied everywhere, even if it is suboptimal at each site. The fourth is feasibility: The protocol must be doable given the available resources. The remaining three criteria are flexibility, transparency, and accountability, which mean that protocols should be adaptable to resources, clearly explained for replication and appropriate for evaluation (Cardoso, 2009). Here, we address each criterion and suggest a repeatable, flexible, transparent, and accountable method to design efficient, suitable, comparable, and feasible sampling protocols.

Cardoso (2009) developed protocols to sample in an optimized and standardized way—to obtain the most information possible with a given level of effort—by applying an optimization algorithm to existing data and by selecting a combination of samples that is as efficient as possible across all sites. This approach for creating protocols is flexible: Different nested subprotocols with varying levels of effort may be defined to cope with different objectives or available resources (human, time, or financial). This algorithm is applicable to any taxon and method, and in any biome, as long as the effort per sample is comparable. This comparability can be ensured by measuring effort using person‐hours as the unit of quantitative sampling, which has been applied to spiders in many habitats, for example temperate forests (Coddington, Young, & Coyle, 1996; Dobyns, 1997; Scharff, Coddington, Griswold, Hormiga, & de Bjørn, 2003); habitats with no tree cover (Carvalho et al., 2012; Toti, Coyle, & Miller, 2000); savanna (Muelelwa, Foord, Dippenaar‐Schoeman, & Stam, 2010); and tropical forests (Coddington, Griswold, Silva Dávila, Peñaranda, & Larcher, 1991; Silva‐Davila & Coddington, 1996). However, the question remains as to whether such protocols—and their development—are applicable effectively to extremely diverse communities.

Here, we show that optimized and standardized protocols for collecting quantitative data on communities (species composition and relative abundance) are not only easy to develop and more informative than traditional nonoptimized protocols, but also feasible even for megadiverse taxa, such as tropical spiders. Spiders are an excellent test group for developing effort‐focused standard protocols because they are speciose and abundant both locally and worldwide in terrestrial ecosystems (Basset et al., 2012; World Spider Catalog, 2016). With many small, cryptic, and locally rare species, it is unrealistic to compile complete species lists for most habitats, even more so in high‐diversity habitats such as tropical forests.

From an ecological and conservation perspective, spiders provide valuable information as they are especially sensitive to habitat disturbance (Malumbres‐Olarte, Vink, Ross, Cruickshank, & Paterson, 2013). Because they are usually dominant invertebrate predators, spiders are also potential indicators of trends in the populations of taxa—such as their prey—that may take longer to go extinct (Cardoso, Arnedo, Triantis, & Borges, 2010). Conservation studies often lack reliable invertebrate data, but the wide adoption of standard protocols can help to solve this problem (Cardoso, Pekár, Jocqué, & Coddington, 2011).

Our objectives are (1) to develop the first optimized and standardized sampling protocol for tropical spider communities using a large body of existing data; (2) to evaluate the efficiency of this protocol by comparing it with two alternatives: protocols tailored to each site following expert opinion (a commonly used approach and here termed ad hoc) and protocols that are statistically optimal for each individual site (termed *optimal*) (see definitions in Materials and Methods); and (3) to test the effectiveness and feasibility of our protocol in the field.

## 2 Materials and Methods

In this study, a “standardized protocol” is defined as a protocol that is designed to be applicable to all sites of the same habitat type and to provide data comparable across sites. In contrast, an “optimized protocol” distributes the number of samples among methods to obtain the theoretical maximum possible number of species, and provides as much information on species assemblages (composition and relative abundances) as possible with minimum effort. A protocol can be optimized either for only a specific site (“optimal protocol”) or for multiple sites (“quasi‐optimal protocol”). A quasi‐optimal protocol is therefore standardized and may not be optimal for any specific site alone. Here, the quasi‐optimal protocol for tropical forests is named COBRA‐TF (Conservation Oriented Biodiversity Rapid Assessment for Tropical Forests) after Cardoso (2009).

Three protocols are compared: (1) quasi‐optimal (COBRA‐TF) (standardized, optimized); (2) ad hoc (not standardized, not optimized); and 3) optimal (not standardized, optimized). Ad hoc protocols are site specific and based on little or no quantitative analysis of sample data—designed according to the best judgment of experienced collectors as to what combination of methods would provide the maximum number of species.

### 2.1 Protocol design

**Figure 1:**
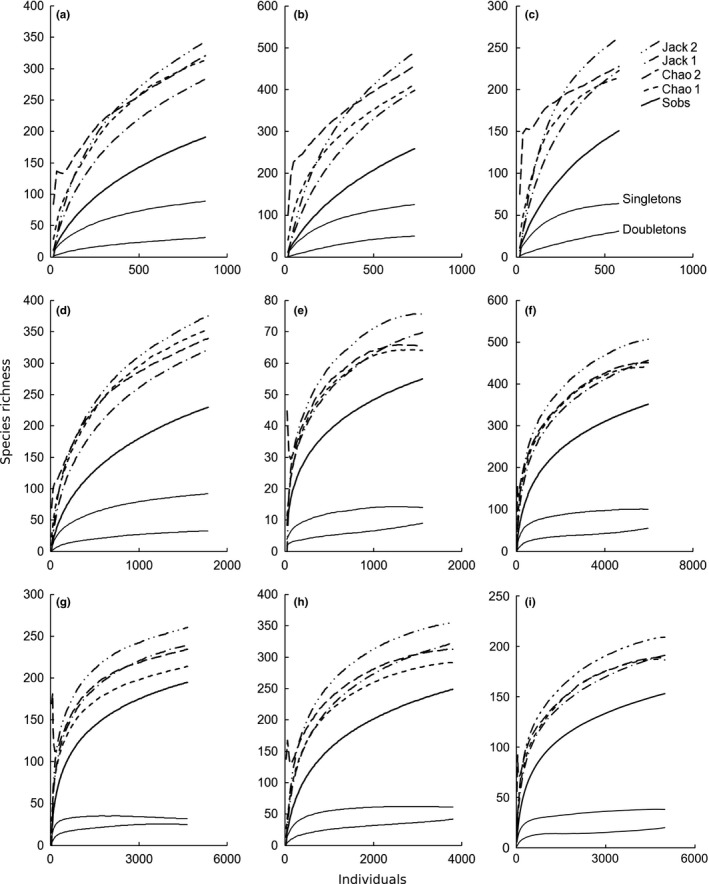
Accumulation curves of observed species richness, estimated species richness, and singletons and doubletons of the sampling sites in (a) Bolivia (Estación Biologica Beni); (b) Bolivia (Rio Tigre); (c) Bolivia (Cerro Uchumachi); (d) Cameroon (Etome); (e) Cameroon (Mann Springs); (f) Guyana; (g) Madagascar (Montagne D'Abre); (h) Madagascar (Marojezy); (i) Tanzania

The COBRA‐TF was developed using data from semiquantitative spider inventories from tropical forests in South America and Africa (Table 1 and Figure 1). Nine studies where sampling was conducted in a one‐hectare (100 × 100 m) plot were considered. Although the collecting teams had different numbers of collectors and the numbers of samples per collecting method were specifically designed for each site (ad hoc), individual samples were standardized to one hour to be comparable from site to site. Six sampling methods were used during the day (d) and night (n), and each combination was considered independent in the analyses, giving a total of 12 separate methods (Cardoso, 2009; Coddington, Agnarsson, Miller, Kuntner, & Hormiga, 2009; Sørensen, Coddington, & Scharff, 2002):

**Table 1:** Sites considered for designing the COBRA‐TF protocol with distribution of samples, abundance, richness, and estimated richness

Country (Area name)	Bolivia (Estacion Biologica Beni)^a^	Bolivia (Rio Tigre) a	Bolivia (Cerro Uchumachi) a	Cameroon (Etome) b	Cameroon (Mann Springs) b	Guyana (Upper Takutu‐Essequibo) c	Madagascar (Montagne D'Abre) b	Madagascar (Marojezy) b	Tanzania (Masisiwe, Udzungwa Mts) d
Number of samples	51	45	32	125	71	300	152	139	220
Ad	1	1	2	14	5	12	13	13	7
An	15	9	7	26	22	76	28	31	41
Bd	15	19	6	35	14	36	24	21	16
Bn	0	2	0	5	4	19	14	5	33
Cd	0	0	0	17	7	28	10	13	25
Cn	0	0	0	0	0	20	6	2	20
Gd	5	8	14	12	7	28	15	21	4
Gn	15	6	3	16	12	32	18	10	37
Sd	0	0	0	0	0	2	8	5	16
Sn	0	0	0	0	0	1	0	0	1
Pf	0	0	0	0	0	46	16	18	20
Individuals (*n*)	875	732	579	1,780	1,555	5,965	4,641	3,797	4,987
Observed species richness (S)	191	259	151	230	55	352	195	249	153
Sampling intensity (*n*/S)	5	3	4	8	28	17	24	15	33
Singletons	89 (47%)	126 (49%)	64 (42%)	92 (40%)	14 (25%)	101 (29%)	32 (16%)	61 (24%)	38 (25%)
Doubletons	31 (16%)	50 (19%)	31 (21%)	33 (14%)	9 (16%)	56 (16%)	25 (13%)	42 (17%)	20 (13%)
Chao1 ± SD (S*)	313 ± 35	413 ± 37	214 ± 21	353 ± 35	64 ± 6	441 ± 23	214 ± 9	292 ± 14	187 ± 10
Chao2 ± SD	321 ± 37	458 ± 46	228 ± 24	339 ± 31	65 ± 7	451 ± 26	234 ± 15	313 ± 20	188 ± 8
Jackknife1 ± SD	283 ± 13	399 ± 16	223 ± 12	321 ± 11	70 ± 5	457 ± 10	240 ± 9	322 ± 10	191 ± 10
Jackknife2	343	490	262	376	76	507	261	355	209
Sampling completeness (S/S*)	61%	63%	71%	65%	86%	80%	91%	85%	91%
Slope S*	0.092	0.290	0.059	0.079	−0.001	−0.002	0.007	0	0.007

Sampling completeness and slope values were calculated based on the Chao1 estimator (Ad/n—aerial day/night; Bd/n—beating day/night; Cd/n—cryptic day/night; Gd/n—ground day/night; Sd/n—sweeping day/night; Pf—pitfall).

a Coddington et al. (1991).

b Coddington, J.A., Griswold, C.E., Hormiga, G., Larcher, S.F. (unpublished data).

c Coddington et al. (2009).

d Sørensen et al. (2002).

Aerial hand collecting (aerial, Ad/An)—hand collecting from knee level to as high as one can reach. This method targets web‐building and/or free‐living spiders on the foliage and stems of living or dead shrubs, high herbs, tree trunks, or lianas.
Ground hand collecting (ground, Gd/Gn)—hand collecting of spiders visible on (but not hiding in) the leaf litter and on the ground, low buttresses, logs, and the lowest vegetation. It covers the vegetation stratum from ground to knee level.
Cryptic searching (cryptic, Cd/Cn)—hand collecting of species hiding in cryptic habitats (e.g., within litter, small holes in trees or fallen logs, under logs, bark, stones, and moss) or litter sampling performed either by direct search, or search in unsifted/sifted litter.
Vegetation beating (beating, Bd/Bn)—beating the vegetation with a rigid stick while holding a beating tray or screen underneath, from which the spiders were collected (Coddington et al., 1996). This method collects spiders living in shrubs, high herb vegetation, bushes, and lower branches of trees.
Sweep netting (sweeping, Sd/Sn)—sweeping low, primarily herbaceous or shrubby vegetation using a sweep net. The net was emptied at regular intervals (after three to five sweeps) to avoid loss or destruction of the specimens.
Pitfall trapping (pitfall, Pt)—pitfalls 9 cm in diameter, partly filled with preservative solution and a few drops of liquid soap to break the surface tension, and sheltered by lids on stilts 2–3 cm above ground. Traps were left in the field for 5–8 days. Groups of five pitfall samples were pooled to reduce variation in the abundance of adult spiders between samples and to make pitfall samples comparable to one person‐hour effort—it takes around an hour to dig and fill five traps and to collect them after the sampling period.

All samples (except pitfall traps) comprised of one hour of continuous active sampling, measured with a stopwatch. Activity not directly involved in sampling was excluded by pausing the stopwatch (e.g., travel time to a different area within the plot, logistical problems). Aspirators were generally used to transfer small specimens to vials. All putatively adult spiders seen were collected and transferred to vials with ethanol.

All adult specimens were identified at least to family level and sorted to species or morphospecies (as the majority of the species were undescribed) by examination of genitalia (Oliver & Beattie, 1996). Somatic features, co‐occurrence, and relative abundance were used to match sexes. Once the data were obtained, four steps were followed:

1. *Assessment of the source data*. It is critical to have comprehensive and robust data to develop the protocol and to understand how to select them. Data must come from exhaustive sampling—they must have enough samples per method, and high enough sampling intensity (Coddington et al., 1996) and completeness (Scharff et al., 2003; Sørensen et al., 2002). Sampling completeness was compared using randomized accumulation curves of observed as well as estimated species richness as calculated with Chao1, Chao2, and first‐ and second‐order Jackknife. The “final” slopes of Chao1 curves were used to determine whether an asymptote was reached (Cardoso, Pekár et al., 2011) and was calculated as:
  $$\text{Slope}=\left(S_{a}^{*}-S_{a-1}^{*}\right)/\left(n_{a}-n_{a-1}\right)$$
  where $S_{a}^{*}$ = estimated total number of species; $S_{a-1}^{*}$ = number of estimated species after adding the next to last sample; *n*_a_ = total number of individuals; *n*_a−1_ = number of individuals after adding the next to last sample. Slopes below 0.01 were considered asymptotic. Likewise, the percentages of singletons and doubletons (species represented by one and two specimens, respectively), and the accumulation curves of singletons and doubletons (Coddington et al., 1996; Scharff et al., 2003; Sørensen et al., 2002) were assessed.
2. *Optimization of the effort per collecting method*. The number of samples per method that maximized the species richness for the overall number of samples per site was determined using only the data from sites selected in the previous step. To achieve this, an iterative procedure was followed to maximize the slope of the accumulation curve for any total number or combination of samples as samples were successively added (Cardoso, 2009). This procedure was carried out for all selected sites simultaneously by running 10,000 simulations using an algorithm (function “optim.alpha”) included in the R package BAT (Cardoso, Rigal, & Carvalho, 2015). The minimum number of samples per method was set equal to that used in the 24‐sample protocol for Mediterranean forest (Cardoso, 2009) to ensure comparability with sites worldwide using the original COBRA protocol, a process termed constrained optimization (Cardoso, Carvalho, Crespo, & Arnedo, 2016). In order to include night sweeping in the analysis, empty samples of this method were added to the tropical data so that each site would have at least two samples, as these are part of the original COBRA.
3. *Definition of the overall effort (stop‐rules)*. A sampling completeness of 50% on average for all sites was chosen as a target because this threshold has been applied in different regions, and it was considered reasonable given the high species diversity in tropical forests (Cardoso, 2009; Coddington et al., 2009). The total number of samples per protocol was set to a multiple of six because this is the maximum number of samples one can collect per day without a decline in sample quality due to fatigue (Coddington et al., 1996).
4. *Standardization of the protocol*. Finally, it was checked that the resulting combinations of samples were feasible and practical (e.g., exclusive night sampling is not practical logistically).

### 2.2 Evaluation of protocol efficiency

The COBRA‐TF was evaluated by comparing it with (1) ad hoc protocols, composed of samples chosen randomly from the pool of each site, with numbers of samples per method proportional to the overall sampling; and (2) optimal protocols, where the optimization algorithm was run for each site separately. For each site, the following results of the protocols were compared: observed species and family richness, estimated species richness, phylogenetic and functional diversity, and species abundance distribution.

Species richness was estimated using Chao1 and the abundance‐based coverage estimator (ACE) (Chao & Lee, 1992; Magurran, 2004), two of the most widely used bias‐corrected estimators. Phylogenetic diversity was calculated using a phylogenetic family tree with equal tree branch lengths generated using the latest spider phylogenies (Coddington & Levi, 2005; Dimitrov et al., 2016; Garrison et al., 2016). A functional tree classifying spider families into predatory guilds built using UPGMA with Gower distance (Cardoso, Pekár et al., 2011) was used to compute functional diversity. The structures of the communities were compared by looking at the species abundance distributions obtained using the Gambin model (Matthews et al., 2014; Ugland et al., 2007). Gambin outperforms other models, such as log‐series or lognormal, and its single variable α describes the “dimensionality” of the communities.

For each site, 1,000 sampling simulations were ran, and the confidence intervals of the procedures above were calculated as the 0.025 and 0.975 percentiles of the values reached by each simulation. All analyses were conducted using the programming environment R (R Development Core Team 2015), particularly the package BAT (Cardoso et al., 2015).

### 2.3 Protocol field test

The COBRA‐TF protocol was tested in a tropical montane forest in the Udzungwa Mountains National Park, Tanzania (above Sanje village: 7.7652778°S, 36.8905556°E). The COBRA‐TF was applied in two plots, one at 650 m.a.s.l. (Plot 1) and the other at 850 m.a.s.l (Plot 2). The plot size was 0.25 ha (50 × 50 m) due to the difficulty of finding one‐ha plots sufficiently level and flat for sampling. Our team of two experienced spider collectors and one experienced entomologist conducted the fieldwork during 3 weeks between January and February 2014, in the light rainy season. The collecting methods were used as described above except for the pitfall samples, which were composed of four traps and were collected after 14 days in the field. This modification was needed to make these results comparable to the extensively used COBRA protocol for Mediterranean ecosystems (Cardoso et al., 2009) in order to allow comparisons of spider assemblages at larger scales (results will be reported elsewhere).

The efficiency of the optimized and standardized protocols was assessed through three questions: (1) How steep are the species accumulation curves in two test tropical forest sites? (2) How do sampling completeness and the number of singletons and doubletons compare with those of other combinations? and (3) What is the contribution of each method to the collected species assemblage?

## 3 Results

### 3.1 Protocol design

1. *Assessment of the source data*. Of the nine initial datasets, only four were appropriate for designing the COBRA‐TF protocol: Guyana, Madagascar (two), and Tanzania (Table 1). These sites showed high sampling intensity (above 15) and completeness (above 80%) and relatively low proportions of singletons (below 30%). The accumulation curves for the Chao1 estimator mostly reached an asymptote (slope < 0.008 in all cases), and the singleton curves reached an asymptote or decreased and approached the doubleton curves by the end of the accumulation process (Figure 1). Sweeping was excluded from the analyses of accumulation curves because only a few samples were taken at some sites.
2. *Optimization of the effort per method*. The curves of the optimal protocols were steeper (more efficient) than the curves of the ad hoc protocols at all the sites (Figure 2). Most methods in isolation had less steep slopes than any combination of methods; however, in some cases, aerial night searching and both day and night beating also produced steep curves, especially at the beginning (Figure 2).

**Figure 2:**
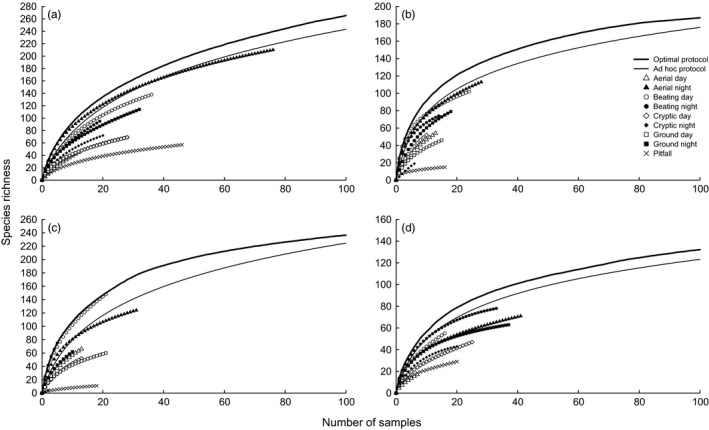
Randomized species accumulation curves of the two site‐specific protocols (optimal and ad hoc) and all individual sampling methods at the four sites selected as thoroughly sampled: (a) Guyana, (b) Madagascar (Montagne D'Abre); (c) Madagascar (Marojezy); (d) Tanzania. Only the first 100 samples are presented
3. *Definition of the overall effort (stop‐rules)*. For the four analyzed areas, 36 samples provided between 41.5% and 63.1% of the species observed in each area and between 33.1% and 57.5% of the estimated species richness (sampling completeness) (Table 2). Being a multiple of six, 36 samples is a pragmatic goal that is possible to attain by a team of two collectors in 3 days or a team of three in 2 days.

**Table 2:** Combination of samples per method of the optimal protocols for each site given 36 samples per site

	Guyana	Madagascar (MdA)	Madagascar (Mj)	Tanzania
Ad	0	0	0	0
An	22	7	4	8
Bd	6	11	21	16
Bn	0	5	5	0
Cd	4	7	4	5
Cn	2	0	0	0
Gd	0	0	1	0
Gn	1	6	1	5
Pf	1	0	0	2
%S	41.5	63.1	59.8	48.2
%S*	33.1	57.5	51	42.1

See also the percentages of species with respect to the observed (%S) and the estimated (%S*) number of species in all samples in each site.
4. *Standardization of the protocol*. In general, optimal protocols were highly biased toward aerial night and beating day sampling, with some samples of either day or night cryptic and ground sampling (Table 2). Pitfall trapping was seldom chosen by the algorithm. The ideal compromise protocol across all sites includes a large proportion of aerial night and beating day sampling and small proportions of both day and night ground searching.

Using the Mediterranean forest COBRA as the base for COBRA‐TF and optimizing all sites simultaneously resulted in a different combination of samples, mainly due to the inclusion of pitfall traps. Numbers were kept even to make them easier to apply in the field. Also, ground hand collecting and cryptic searching were combined in a single sampling method that can be adjusted to the features of the ground—depending on the ground cover and the amount of dead logs or rocks, more time may be spent using either method. The final combination of samples was 8 An + 6 Bd + 2 Bn + 2 Gd/Cd + 2 Gn/Cn + 2 Sd + 2 Sn + 12 Pt.

### 3.2 Evaluation of protocol efficiency

**Figure 3:**
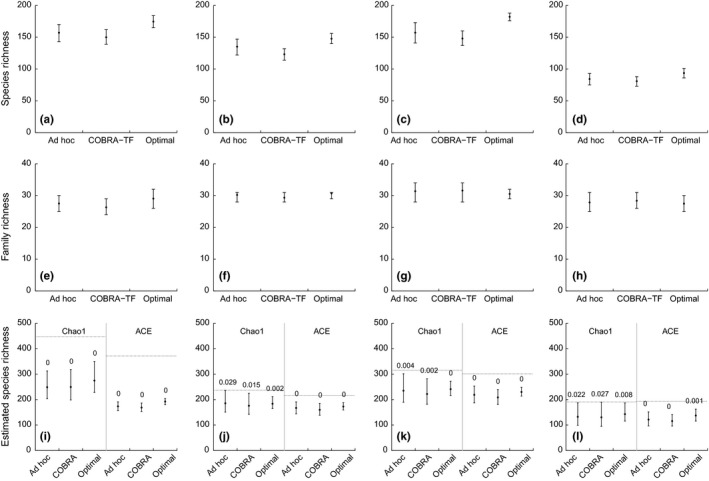
Observed species (a–d) and family (e–h), and estimated species (i–l) richness obtained from the ad hoc, COBRA–TF and optimal protocols. Results are based on 1,000 simulated protocols with 36 samples for Guyana (a, e), Madagascar (Montagne D'Abre) (b, f), Madagascar (Marojezy) (c, g), and Tanzania (d, h). In graphs (i–l), each site shows the means (dots), 95% confidence intervals (bars), and the *p* values between the Chao1 and ACE estimates of the full dataset (broken horizontal lines) and the four estimates (percentage of values from the simulations larger than the mean of the full‐dataset estimate)

**Figure 4:**
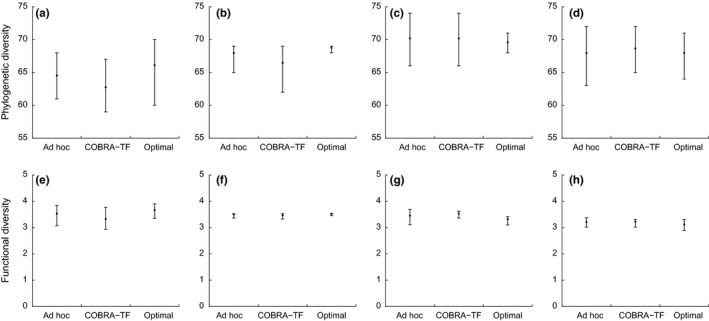
Phylogenetic (a–d) and functional (e–h) diversity measures obtained from the ad hoc, COBRA–TF, and optimal protocols. Results are based on 1,000 simulated protocols with 36 samples for Guyana (a, e), Madagascar (Montagne D'Abre) (b, f), Madagascar (Marojezy) (c, g), and Tanzania (d, h)

**Figure 5:**
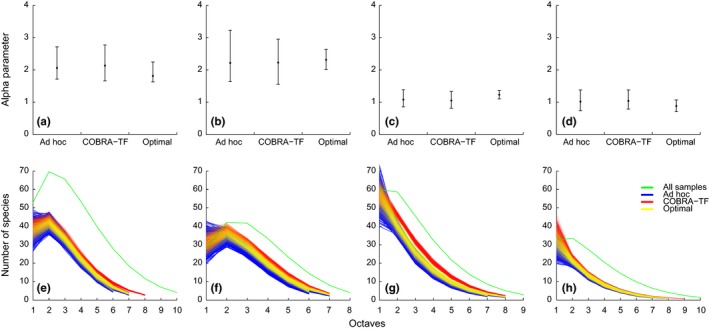
Alpha parameter (a–d) and species abundance distributions using the Gambin model (e–h) obtained from the ad hoc, COBRA–TF, and optimal protocols. Results are based on 1,000 simulated protocols with 36 samples for Guyana (a ,e), Madagascar (Montagne D'Abre) (b ,f), Madagascar (Marojezy) (c, g), and Tanzania (d, h)

Optimal protocols yielded more observed species than the COBRA‐TF and the ad hoc protocols (Figure 3), which were similar. All protocols provided similar estimated number of species but underestimated the true total species richness (Figure 3). Family richness (Figure 3) and phylogenetic and functional diversity (Figure 4) were similar across protocols. The α parameter of the species abundance distribution models was similar and the curves of the Gambin model overlapped in all sites (Figure 5).

### 3.3 Protocol field test

**Figure 6:**
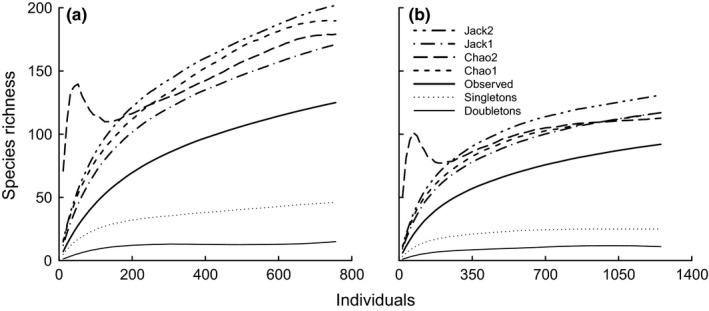
Species accumulation curves obtained from the field test of the COBRA‐TF protocol. Lines represent observed species richness, estimated species richness, and singletons and doubletons of test plots 1 (a) and 2 (b)

In the test plots 1 and 2, 756 and 1,252 adult specimens belonging to 125 and 92 morphospecies, respectively (Table 3), were collected. The final slopes of Chao1 estimators were above 0.03 (Figure 6 and Table 3) and sampling completeness was 65.9% and 78.6% (Table 3), although these numbers are probably overestimates (Figure 6). The percentage of singletons was 37% and 27% and the percentage of doubletons 12% for both plots. Sampling intensity was relatively low (Table 3): 6.1 and 13.6.

**Table 3:** Quantitative data from the field test of the COBRA‐TF protocol. Estimates were calculated as in Table 1

	Plot 1 (low)	Plot 2 (top)
Individuals (*n*) (36)	756	1,252
Observed species richness (S)	125	92
Sampling intensity (*n*/S)	6.1	13.6
Singletons	46 (37%)	25 (27%)
Doubletons	15 (12%)	11 (12%)
Chao1 (S*)	190 ± 28	117 ± 9
Chao2	179 ± 11	113 ± 3
Jackknife1	171 ± 18	117 ± 10
Jackknife2	202 ± 34	131 ± 14
Sampling completeness (S/S*)	65.9%	78.6%
Slope S*	0.0399	0.0316

Aerial night sampling resulted in the largest percentage of unique species among methods, whereas sweeping day and night provided the largest number of species per sample (Table 4). These two methods plus beating made the biggest contribution per sample in terms of unique species. Ground collecting and, particularly, pitfall trapping had the lowest numbers of species as well as unique species per sample (Table 4).

**Table 4:** Number of species and percentages of total species (%) collected per method in the field test of the COBRA‐TF protocol

Method	Unique species per method		Mean number of species per sample		Mean number of species unique to method per sample	
	Plot 1	Plot 2	Plot 1	Plot 2	Plot 1	Plot 2
An	24 (19.2)	17 (18.5)	18.1 (14.5)	15.3 (16.6)	6.9 (5.5)	4.1 (4.5)
Bd	11 (8.8)	3 (3.3)	18 (14.4)	16.5 (17.9)	4.5 (3.6)	5 (5.4)
Bn	1 (0.8)	3 (3.3)	20 (16)	19 (20.7)	8 (6.4)	7 (7.6)
Gd	2 (1.6)	1 (1.1)	9 (7.2)	11 (12)	4 (3.2)	2 (2.2)
Gn	1 (0.8)	0 (0)	11.5 (9.2)	11 (12)	4 (3.2)	2 (2.2)
Pt	3 (2.4)	8 (8.7)	3.4 (2.7)	4.7 (5.1)	1.5 (1.2)	1.8 (2)
Sd	10 (8)	3 (3.3)	26 (20.8)	21.5 (23.4)	7.5 (6)	6 (6.5)
Sn	3 (2.4)	1 1.1)	24.5 (19.6)	21.5 (23.4)	6 (4.8)	6 (6.5)

## 4 Discussion

Optimized and standardized protocols are preferable to ad hoc protocols for two major reasons. First, they are equally efficient as measured in person‐hours of work needed to achieve a given sampling completeness or to provide data on the phylogenetic and functional diversity, and the species abundance distribution in a community. Second, they enable comparison of data from other studies and areas without having to account for sampling effort in the analyses. Not only does this help answer questions at larger scales but it allows reusing the data beyond the objectives of each particular project. The data resulting from the new protocol can now be easily and fully compared with previous data collected in different regions for multiple purposes such as inventorying (Crespo, Cardoso, Carvalho, Henriques, & Rufino, 2009), conservation (Crespo, Silva, Borges, & Cardoso, 2013), or biogeography (Carvalho et al., 2011a,b, 2012).

Our results show that a protocol combining day and night sampling is necessary to ensure an efficient coverage of the spider diversity at tropical forest sites. We propose that the combination of samples of COBRA‐TF be the minimum so that if a more comprehensive protocol is applied, the resulting data are still comparable after subsampling from the minimum common denominator. Surprisingly, pitfall traps, usually deployed in the field during relatively long periods, are not particularly efficient in tropical forests, contrary to what has been found in temperate areas (Cardoso, Gaspar et al., 2008; Cardoso, Scharff et al., 2008; Cardoso et al. 2009). Dense ground vegetation may act as obstacles, reducing movement and decreasing capture of ground active spiders by pitfall traps (Malumbres‐Olarte et al., 2013). More complex ground vegetation in tropical forests as well as greater competition with other predators such as ants may explain our results, although corroborating this hypothesis requires specific tests. Nevertheless, including pitfalls in the COBRA‐TF protocol does enable comparison with previous implementations of COBRA, and pitfall traps collect ground and leaf‐litter species that are missed by other methods (Cardoso, Pekár et al., 2011). Moreover, these samples often contain more endemic species than arboreal samples, as arboreal spiders, especially web‐building species, usually have higher dispersal ability through ballooning (Larrivée & Buddle, 2011).

All estimates and conclusions derived from COBRA‐TF are limited to the lower vegetation and ground and thereby miss an important component of tropical diversity: the canopy fauna (Moffett, 1994). Canopy fogging, or other methods targeting the high vegetation, is essential to obtain a complete representation of spider species in tropical forests (Fannes, De Bakker, Loosveldt, & Jocqué, 2008; Sørensen, 2004). However, fogging is logistically demanding and as it is not time based, it may be difficult to combine with the quantifiable methods used in the analyses.

Based on our field test of COBRA‐TF, we provide a number of recommendations adaptable to taxa other than spiders. First, we recommend limiting the number of one‐hour samples per collector to six to avoid reduced sampling quality due to fatigue (Cardoso, 2009; Coddington et al., 1996). Second, we suggest concurrent collecting by multiple teams in different plots when logistics and available resources allow it. While requiring more resources, this will save time overall, and will reduce the effects of species phenology, rapid changes in community composition or capture efficiency due to varying weather conditions. Third, we suggest reducing sampling area from the originally proposed 1 ha to 0.25 ha (50 × 50 m) because finding multiple suitable areas of 1 ha can be a difficult task in topographically complex and fragmented habitats of montane tropical forests. The reduction in area should not cause any measurable effect on the number of species captured with low‐effort sampling, as effort appears to be overwhelmingly more important than area (Coddington et al., 2009; Sørensen et al., 2002). Fourth, collecting should be conducted when vegetation is dry, when most collecting methods are more efficient. Although collecting in the rainy season may yield more species than in the dry season (Azevedo et al., 2014), it may be more time‐consuming and therefore eventually become more expensive—in our field test, two to three collectors needed 4–5 days to collect 24 hand‐collected samples because of intermittent rain during the light rainy season. A possible compromise and our recommendation for maximum efficiency is to collect shortly after the rainy season. Finally, we warn about the potential effects of simultaneous or continuous sampling in the same small area even with low sampling effort, that is, the number and identity of the specimens collected in an aerial sample may be different if a beating sample has been recently collected from the same vegetation. Because of this, we suggest sampling systematically to cover as much of the plot as possible, minimizing concurrent sampling and postponing more vegetation‐damaging methods (e.g., beating, sweeping) to follow the other methods.

The COBRA‐TF and the ad hoc protocols are similar in estimating species richness using nonparametric estimators with low number of samples. Currently available species richness estimators often require comprehensive sampling to reach an asymptote indicative of a reliable estimate (Cardoso, Rigal, Borges, & Carvalho, 2014; Walther & Morand, 1998), so less “data‐hungry” and less biased estimators are needed. In tropical forests and for megadiverse groups, accurate estimates require a large sampling effort, involving several collectors in a single site during one or more weeks (Coddington et al., 2009), and such effort, although possible, is rarely made in most studies. Estimates are often required when comparing assemblages using incomplete species lists, but this need may be avoided if optimized and standardized sampling is used.

Here, we prove that optimized and standardized protocols such as COBRA‐TF can be developed for megadiverse taxa and that their use is not restricted to less diverse and, in principle, more easily quantifiable communities, such as those in Mediterranean and temperate habitats. COBRA‐TF is as good as traditional ad hoc protocols at collecting data despite the fact that COBRA‐TF is based on data from only four sites. Designing a perfectly universal sampling protocol for tropical forest would require data from dozens of sites from all over the world, but such data are not available yet. Nevertheless, our field test proves that the COBRA‐TF performs adequately in other tropical forests and is a sound protocol that may be refined through constant testing and addition of data from new sites.

Comparability, short duration and easy application make the COBRA‐TF protocol extremely useful. But above all, adaptability is the key characteristic: The approach of COBRA‐TF—the process of optimization and standardization—is applicable to most megadiverse taxa, habitats, and sampling methods. For instance, following the optimization and standardization steps outlined above and using data collected in locations with similar habitat conditions, one could develop a COBRA protocol for highly diverse tropical forest amphibians: an optimized combination of samples of methods such as time‐standardized dip netting, and number‐standardized PVC tubes and pitfall trapping. Likewise, a protocol for megadiverse Lepidoptera communities may consist of light trap and Malaise trap samples standardized per time.

If we are to record, quantify, and assess some of the most diverse and unique communities in the world, we must apply efficient, widely applicable, and standardized tools, such as COBRA‐TF. Not only will they allow comparing communities in order to understand the ecological processes behind their assembly, they will also facilitate monitoring and assessing megadiverse communities, their changes in space and time caused by climatic changes or human disturbances, and selecting the areas optimizing their conservation.

## Data Accessibility

All the species abundance data used in the analyses will be available from the CMEC Research Data Archive (University of Copenhagen).

## Conflict of Interest

None declared.

## References

[ece32626-bib-0001] Agosti, D. , Majer, J. D. , Alonso, L. E. , & Schultz, T. R. eds. (2000). Ants: Standard methods for measuring and monitoring biodiversity. (pp. 280). Washington and London: Smithsonian Institution Press.

[ece32626-bib-0002] Azevedo, G. H. F. , Faleiro, B. T. , Magalhães, I. L. F. , Benedetti, A. R. , Oliveira, U. , Pena‐Barbosa, J. P. P. , ··· Santos, A. J. (2014). Effectiveness of sampling methods and further sampling for accessing spider diversity: A case study in a Brazilian Atlantic rainforest fragment. Insect Conservation and Diversity, 7, 381–391.

[ece32626-bib-0003] Basset, Y. , Cizek, L. , Cuénoud, P. , Didham, R. K. , Guilhaumon, F. , Missa, O. , ··· Leponce, M. (2012). Arthropod diversity in a tropical forest. Science (New York, NY), 338, 1481–1484. 10.1126/science.1226727 23239740

[ece32626-bib-0004] Blackmore, S. (1996). Knowing the Earth's biodiversity: Challenges for the infrastructure of systematic biology. Science, 274, 63–64.

[ece32626-bib-0005] Cardoso, P. (2009). Standardization and optimization of arthropod inventories‐the case of Iberian spiders. Biodiversity and Conservation, 18, 3949–3962.

[ece32626-bib-0006] Cardoso, P. , Arnedo, M. A. , Triantis, K. A. , & Borges, P. A. V. (2010). Drivers of diversity in Macaronesian spiders and the role of species extinctions. Journal of Biogeography, 37, 1034–1046.

[ece32626-bib-0007] Cardoso, P. , Carvalho, J. C. , Crespo, L. C. , & Arnedo, M. A. (2016). Optimal inventorying and monitoring of taxon, phylogenetic and functional diversity. Biorxiv, 1–18. doi: http://dx.doi.org/10.1101/060400 10.1371/journal.pone.0307156 PMC11290677 39083565

[ece32626-bib-0008] Cardoso, P. , Crespo, L. C. , Carvalho, R. , Rufino, A. C. , & Henriques, S. S. (2009). Ad‐Hoc vs. standardized and optimized arthropod diversity sampling. Diversity, 1, 36–51.

[ece32626-bib-0009] Cardoso, P. , Erwin, T. L. , Borges, P. A. V. , & New, T. R. (2011). The seven impediments in invertebrate conservation and how to overcome them. Biological Conservation, 144, 2647–2655.

[ece32626-bib-0010] Cardoso, P. , Gaspar, C. , Pereira, L. C. , Silva, I. , Henriques, S. S. , da Silva, R. R. , & Sousa, P. (2008). Assessing spider species richness and composition in Mediterranean cork oak forests. Acta Oecologica‐International Journal of Ecology, 33, 114–127.

[ece32626-bib-0011] Cardoso, P. , Pekár, S. , Jocqué, R. , & Coddington, J. A. (2011). Global patterns of guild composition and functional diversity of spiders. PLoS ONE, 6, e21710. 21738772 10.1371/journal.pone.0021710 PMC3126856

[ece32626-bib-0012] Cardoso, P. , Rigal, F. , Borges, P. A. V. , & Carvalho, J. C. (2014). A new frontier in biodiversity inventory: A proposal for estimators of phylogenetic and functional diversity. Methods in Ecology and Evolution, 5, 452–461.

[ece32626-bib-0013] Cardoso, P. , Rigal, F. , & Carvalho, J. C. (2015). BAT – Biodiversity Assessment Tools, an R package for the measurement and estimation of alpha and beta taxon, phylogenetic and functional diversity. Methods in Ecology and Evolution, 6, 232–236.

[ece32626-bib-0014] Cardoso, P. , Scharff, N. , Gaspar, C. , Henriques, S. S. , Carvalho, R. , Castro, P. H. , ··· Crespo, L. C. (2008). Rapid biodiversity assessment of spiders (Araneae) using semi‐quantitative sampling: A case study in a Mediterranean forest. Insect Conservation and Diversity, 1, 71–84.

[ece32626-bib-0015] Carvalho, J. C. , Cardoso, P. , Crespo, L. C. , Henriques, S. , Carvalho, R. , & Gomes, P. (2011a). Biogeographic patterns of spiders in coastal dunes along a gradient of mediterraneity. Biodiversity and Conservation, 20, 873–894.

[ece32626-bib-0016] Carvalho, J. C. , Cardoso, P. , Crespo, L. C. , Henriques, S. , Carvalho, R. , & Gomes, P. (2011b). Determinants of beta diversity of spiders in coastal dunes along a gradient of mediterraneity. Diversity and Distributions, 17, 225–234.

[ece32626-bib-0017] Carvalho, J. C. , Cardoso, P. , Crespo, L. C. , Henriques, S. , Carvalho, R. , & Gomes, P. (2012). Determinants of spider species richness in coastal dunes along a gradient of mediterraneity. Insect Conservation and Diversity, 5, 127–137.

[ece32626-bib-0018] Chao, A. , & Jost, L. (2012). Coverage‐based rarefaction and extrapolation: Standardizing samples by completeness rather than size. Ecology, 93, 2533–2547. 23431585 10.1890/11-1952.1

[ece32626-bib-0019] Chao, A. , & Lee, S.‐M. (1992). Estimating the number of classes via sample coverage. Journal of the American Statistical Association, 87, 210–217.

[ece32626-bib-0020] Coddington, J. A. , Agnarsson, I. , Miller, J. A. , Kuntner, M. , & Hormiga, G. (2009). Undersampling bias: The null hypothesis for singleton species in tropical arthropod surveys. Journal of Animal Ecology, 78, 573–584. 19245379 10.1111/j.1365-2656.2009.01525.x

[ece32626-bib-0021] Coddington, J. A. , Griswold, C. E. , Silva Dávila, D. , Peñaranda, E. , & Larcher, S. F. (1991). Designing and testing sampling protocols to estimate biodiversity in tropical ecosystems The unity of evolutionary biology: Proceedings of the Fourth International Congress of Systematic and Evolutionary Biology. pp. 44–66. Portland: Dioscorides Press.

[ece32626-bib-0022] Coddington, J. A. , & Levi, H. W. (1991). Systematics and evolution of spiders (Araneae). Annual Review of Ecology and Systematics, 22, 565–592.

[ece32626-bib-0023] Coddington, J. A. , & Levi, H. W. (2005). Phylogeny and classification of spiders In Ubick D., Paquin P., Cushing P.E., & Roth V. (Eds.), Spiders of North America (pp. 18–24). American Arachnological Society.

[ece32626-bib-0024] Coddington, J. A. , Young, L. H. , & Coyle, F. A. (1996). Estimating spider species richness in a Southern Appalachian cove hardwood forest. Journal of Arachnology, 24, 111–128.

[ece32626-bib-0025] Colwell, R. K. , & Coddington, J. A. (1994). Estimating terrestrial biodiversity through extrapolation. Philosophical Transactions of the Royal Society Series B — Biological Sciences, 345, 101–118. 10.1098/rstb.1994.0091 7972351

[ece32626-bib-0026] Crespo, L. C. , Cardoso, P. , Carvalho, R. , Henriques, S. S. , & Rufino, A. C. (2009). Spiders (Arachnida: Araneae) from the Paúl de Arzila Natural Reserve (Portugal). Boletín de la Sociedad Entomológica Aragonesa, 44, 305–313.

[ece32626-bib-0027] Crespo, L. C. , Silva, I. , Borges, P. A. V. , & Cardoso, P. (2013). Rapid biodiversity assessment, faunistics and description of a new spider species (Araneae) from Desertas Islands and Madeira (Portugal). Revista Ibérica de Aracnología, 23, 11–23.

[ece32626-bib-0028] Didham, R. K. , Basset, Y. , & Leather, S. R. (2010). Research needs in insect conservation and diversity. Insect Conservation and Diversity, 3, 1–4.

[ece32626-bib-0029] Dimitrov, D. , Benavides, L. R. , Arnedo, M. A. , Giribet, G. , Griswold, C. E. , Scharff, N. , & Hormiga, G. (2016). Rounding up the usual suspects : a standard target‐gene approach for resolving the interfamilial phylogenetic relationships of ecribellate orb‐weaving spiders with a new family‐rank classification. Cladistics 1–30. 10.1111/cla.12165 34715728

[ece32626-bib-0030] Dirzo, R. , Young, H. S. , Galetti, M. , Ceballos, G. , Isaac, N. J. B. , & Collen, B. (2014). Defaunation in the Anthropocene. Science, 345, 401–406. 25061202 10.1126/science.1251817

[ece32626-bib-0031] Dobyns, J. R. (1997). Effects of sampling intensity on the collection of spider (Araneae) species and the estimation of species richness. Environmental Entomology, 26, 150–162.

[ece32626-bib-0032] Duelli, P. (1997). Biodiversity evaluation in agricultural landscapes: An approach at two different scales. Agriculture, Ecosystems & Environment, 62, 81–91.

[ece32626-bib-0033] Duelli, P. , Obrist, M. K. , & Schmatz, D. R. (1999). Biodiversity evaluation in agricultural landscapes: Above‐ground insects. Agriculture, Ecosystems and Environment, 74, 33–64.

[ece32626-bib-0034] Fannes, W. , De Bakker, D. , Loosveldt, K. , & Jocqué, R. (2008). Estimating the diversity of arboreal oonopid spider assemblages (Araneae, Oonopidae) at Afrotropical sites. Journal of Arachnology, 36, 322–330.

[ece32626-bib-0035] Garrison, N. L. , Rodriguez, J. , Agnarsson, I. , Coddington, J. A. , Griswold, C. E. , Hamilton, C. A. , ··· Bond, J. E. (2016) Spider phylogenomics: untangling the Spider Tree of Life. PeerJ 4:e1719. 10.7717/peerj.1719 PMC4768681 26925338

[ece32626-bib-0036] Hering, D. , Moog, O. , Sandin, L. , & Verdonschot, P. F. M. (2004). Overview and application of the AQEM assessment system. Hydrobiologia, 516, 1–20.

[ece32626-bib-0037] Jones, D. T. , & Eggleton, P. (2000). Sampling termite assemblages in tropical forests: Testing a rapid biodiversity assessment protocol. Journal of Applied Ecology, 37, 191–203.

[ece32626-bib-0038] Larrivée, M. , & Buddle, C. M. (2011). Ballooning propensity of canopy and understorey spiders in a mature temperate hardwood forest. Ecological Entomology, 36, 144–151.

[ece32626-bib-0039] Lawton, J. H. , Bignell, D. E. , Bolton, B. , Bloemers, G. F. , Eggleton, P. , Hammond, P. M. , ··· Watt, A. D. (1998). Biodiversity inventories, indicator taxa and effects of habitat modification in tropical forest. Nature, 391, 72–76.

[ece32626-bib-0040] Magurran, A. E. (2004) Measuring biological diversity. Oxford: Blackwell Science Ltd.

[ece32626-bib-0041] Magurran, A. E. , & Queiroz, H. (2010). Evaluating tropical biodiversity: Do we need a more refined approach? Biotropica, 42, 537–539.

[ece32626-bib-0042] Malumbres‐Olarte, J. , Vink, C. J. , Ross, J. G. , Cruickshank, R. H. , & Paterson, A. M. (2013). The role of habitat complexity on spider communities in native alpine grasslands of New Zealand. Insect Conservation and Diversity, 6, 124–134.

[ece32626-bib-0043] Matthews, T. J. , Borregaard, M. K. , Ugland, K. I. , Borges, P. A. V. , Rigal, F. , Cardoso, P. , & Whittaker, R. J. (2014). The gambin model provides a superior fit to species abundance distributions with a single free parameter: Evidence, implementation and interpretation. Ecography, 37, 1002–1011.

[ece32626-bib-0044] Moffett, M. W. (1994). The high frontier: exploring the tropical rainforest canopy. Cambridge, MA: Harvard University Press.

[ece32626-bib-0045] Muelelwa, M. I. , Foord, S. H. , Dippenaar‐Schoeman, A. S. , & Stam, E. M. (2010). Towards a standardized and optimized protocol for rapid biodiversity assessments: Spider species richness and assemblage composition in two savanna vegetation types. African Zoology, 45, 273–290.

[ece32626-bib-0047] New, T. R. (1999). Untangling the web: Spiders and the challenges of invertebrate conservation. Journal of Insect Conservation, 3, 251–256.

[ece32626-bib-0048] Niemelä, J. , Kotze, J. , Ashworth, A. , Brandmayr, P. , Desender, K. , New, T. , ··· Spence, J. (2000). The search for common anthropogenic impacts on biodiversity: A global network. Journal of Insect Conservation, 4, 3–9.

[ece32626-bib-0049] Oliver, I. , & Beattie, A. J. (1996). Invertebrate morphospecies as surrogates for species: A case study. Conservation Biology, 10, 99–109.

[ece32626-bib-0050] Pollard, E. , & Yates, T. J. (1993). Monitoring butterflies for ecology and conservation: The British butterfly monitoring scheme. London: Chapman & Hall.

[ece32626-bib-0051] R Core Team (2015). R: A language and environment for statistical computing. Version 3.1.3. Vienna, Austria: R Foundation for Statistical Computing.

[ece32626-bib-0052] Scharff, N. , Coddington, J. A. , Griswold, C. E. , Hormiga, G. , & de Bjørn, P. (2003). When to quit? Estimating spider species richness in a northern European deciduous forest. Journal of Arachnology, 31, 246–273.

[ece32626-bib-0053] Silva‐Davila, D. , & Coddington, J. A. (1996). Spiders of Pakitza (Madre de Dios, Peru): Species richness and notes on community structure In Wilson D. E. & Sandoval A. (Eds.), The biodiversity of Southeastern Peru (pp. 253–311). Washington: Smithsonian Institution.

[ece32626-bib-0054] Sørensen, L. (2004). Composition and diversity of the spider fauna in the canopy of a montane forest in Tanzania. Biodiversity & Conservation, 13, 437–452.

[ece32626-bib-0055] Sørensen, L. L. , Coddington, J. A. , & Scharff, N. (2002). Inventorying and estimating subcanopy spider diversity using semiquantitative sampling methods in an Afromontane forest. Environmental Entomology, 31, 319–330.

[ece32626-bib-0056] Stork, N. E. , Samways, M. J. , & Eeley, H. A. C. (1996). Inventorying and monitoring biodiversity. Trends in Ecology & Evolution, 11, 39–40. 21237758 10.1016/0169-5347(96)81070-6

[ece32626-bib-0057] Toti, D. S. , Coyle, F. A. , & Miller, J. A. (2000). A structured inventory of appalachian grass bald and heath bald spider assemblages and a test of species richness estimator performance. Journal of Arachnology, 28, 329–345.

[ece32626-bib-0058] Ugland, K. I. , Lambshead, P. J. D. , Mcgill, B. , Gray, J. S. , Dea, N. O. , Ladle, R. J. , & Whittaker, R. J. (2007). Modelling dimensionality in species abundance distributions: Description and evaluation of the Gambin model. Evolutionary Ecology Research, 9, 313–324.

[ece32626-bib-0059] Walther, B. A. , & Morand, S. (1998). Comparative performance of species richness estimation methods. Parasitology, 116(4), 395–405. 9585941 10.1017/s0031182097002230

[ece32626-bib-0046] World Spider Catalog . (2016) World Spider Catalog. Natural History Museum Bern. Retrieved from http://wsc.nmbe.ch/. version 17.5, accessed on November 22 2016.

